# Integrated analysis of lncRNA, miRNA and mRNA reveals novel insights into the fertility regulation of large white sows

**DOI:** 10.1186/s12864-020-07055-2

**Published:** 2020-09-14

**Authors:** Huiyan Hu, Qing Jia, Jianzhong Xi, Bo Zhou, Zhiqiang Li

**Affiliations:** 1 Department of Animal Genetics, Breeding and Reproduction, College of Animal Science and Technology, Hebei Agricultural University, Lekai South Street No. 2596, Baoding, 071000 Hebei China; 2 Engineering Research Center for Agriculture in Hebei Mountainous Areas, Baoding, 071000 Hebei China

**Keywords:** Pigs, Fertility, Ovary, Follicular and luteal phases, Competing endogenous RNAs, Regulation networks

## Abstract

**Background:**

Improving sow fertility is extremely important as it can lead to increased reproductive efficiency and thus profitability for swine producers. There are considerable differences in fertility rates among individual animals, but the underlying molecular mechanisms remain unclear. In this study, by using different types of RNA libraries, we investigated the complete transcriptome of ovarian tissue during the luteal (L) and follicular (F) phases of the estrous cycle in Large White pigs with high (H) and low (L) fecundity, and performed a comprehensive analysis of long noncoding RNAs (lncRNAs), mRNAs and micro RNAs (miRNAs) from 16 samples by combining RNA sequencing (RNA-seq) with bioinformatics.

**Results:**

In total, 24,447 lncRNAs, 27,370 mRNAs, and 216 known miRNAs were identified in ovarian tissues. The genomic features of lncRNAs, such as length distribution and number of exons, were further analyzed. We selected a threshold of *P* < 0.05 and |log_2_ (fold change)| ≥ 1 to obtain the differentially expressed lncRNAs, miRNAs and mRNAs by pairwise comparison (LH vs. LL, FH vs. FL). Bioinformatics analysis of these differentially expressed RNAs revealed multiple significantly enriched pathways (*P* < 0.05) that were closely involved in the reproductive process, such as ovarian steroidogenesis, lysosome, steroid biosynthesis, and the estrogen and GnRH signaling pathways. Moreover, bioinformatics screening of differentially expressed miRNAs that share common miRNA response elements (MREs) with lncRNAs and their downstream mRNA targets were performed. Finally, we constructed lncRNA–miRNA–mRNA regulation networks. The key genes in these networks were verified by Reverse Transcription Real**-**time Quantitative PCR (RT-qRCR), which were consistent with the results from RNA-Seq data.

**Conclusions:**

These results provide further insights into the fertility of pigs andcan contribute to further experimental investigation of the functions of these genes.

## Background

Sow fertility is one of the most important economic parameters in the swine industry, and therefore improving reproduction rate can significantly increase the profitability for swine producers. Candidate genes and quantitative trait loci (QTL) associated with reproductive traits, such as total number (of piglets) born (TNB), have been identified in genome-wide association studies (GWAS) in the past [1–6]. Thus far, 377 quantitative trait loci (QTL) for reproductive traits were detected, of which 159 and 129 QTLs for TNB and number born alive (NBA) have already been identified, respectively [7]. Although GWAS results provide important information regarding specific reproductive traits, revealing the complex regulatory mechanism of reproductive performance is still a challenging task. Therefore, the underlying mechanisms of fertility differences in sows are the subject of constant research.

The mammalian genome encodes a high percentage of noncoding transcripts [8]. Two major subsets of noncoding RNAs (ncRNAs) have been identified by high-throughput sequencing: long noncoding RNAs (lncRNAs) and microRNAs (miRNAs) [9]. lncRNAs are a group of regulatory RNAs that are longer than 200 nucleotides and were recently identified in various tissues of human and pigs [10–13]. On the other hand, miRNAs are an abundant class of short (18–24 nucleotides) and highly conserved sequences of endogenous RNAs, which have been extensively studied in many species [14]. Previous studies have prospectively confirmed that ncRNAs (miRNAs and lncRNAs) were considered as crucial players in both humans and animals, and affect various biological functions [12, 15–17]. Moreover, there is substantial evidence supporting that lncRNAs, acting as competing endogenous RNAs (ceRNAs), contribute to the regulation of cardiac fibrosis [18], muscle differentiation [19] and tumorigenesis [20, 21]. According to the hypothesis ofceRNAs [22], Miao et al. (2016) constructed a miRNA–lncRNA–mRNA,which provided a new insight into understanding sheep fertility [23].

The ovary is the most important reproductive organ of sows and is responsible for synthesizing and secreting hormones, which are essential for the maintenance of the normal reproductive cycles and hormone levels. Ovarian folliculogenesis, ovulation and formation and regression of the corpus luteum occur in the ovary, which repeatedly take place over the reproductive life [24] and regulate reproduction in mammals [25]. Previous reports have shown that miRNAs are involved in ovarian processes [24] and regulate fertility [25, 26]. In addition, lncRNAs represent another category of functional RNA molecules. A recent study demonstrated that lncRNAs also play a significant role in the regulation of sheep fertility [23]. While several articles have focused on the lncRNAs expression profile of pig ovarian tissues [27, 28], none of these studies have interpreted regulatory networks for fertility.

In order to improve our understanding of differences in reproductive performance, we constructed and sequenced two different types of cDNA sequence libraries (16 RNA libraries and 16 small RNA cDNA libraries) from ovarian tissue during the follicular and luteal phases of the estrous cycle in Large White pigs with extreme phenotypes (high and low fertility). We aimed to identify potential regulators (lncRNAs and miRNAs) of fecundity in pigs. Importantly, data in this study were integrated in order to reveal novel insights into molecular mechanisms between the high and low fertility in pigs.

## Results

### Characterization of the ovary transcriptome and identification of mRNA and lncRNAs

To identify key differences associated with reproductive efficiency in sows with extreme phenotypes, we constructed 16 cDNA libraries from ovarian tissues during the follicular and luteal phases of the estrous cycle. A total of 1,509,728,794 raw reads were generated from 16 porcine ovary samples. After removing adapter sequences and low-quality sequences, 1,500,044,340 clean reads were retained and used for further analysis. In each sample, the percentage of clean reads ranged from 99.20 to 99.52% (Additional file 1: Table S1–1). In addition, most clean reads were aligned to the reference genome (Sscrofa10.2), accounting for 81.49 to 84.48% (Table S1–2). A total of 1,122,470 transcripts were assembled by Cuffmerge and Scripture [29]. According to the characteristics of lncRNAs, we used four tools (CPC, CNCI, CPAT and PFAM) to discard potential coding transcripts. In the end, 24,447 lncRNA transcripts were identified (Additional file 2: Table S2). These included 6392 anti-sense lncRNAs (26.15%) and 18,055 intergenic lncRNAs (73.85%) (TableS2). In addition, 27,370 mRNAs were identified by mapping Illumina RNA-seqreads (Additional file 3: Table S3).

### microRNA sequencing and identification

**Fig. 1:**
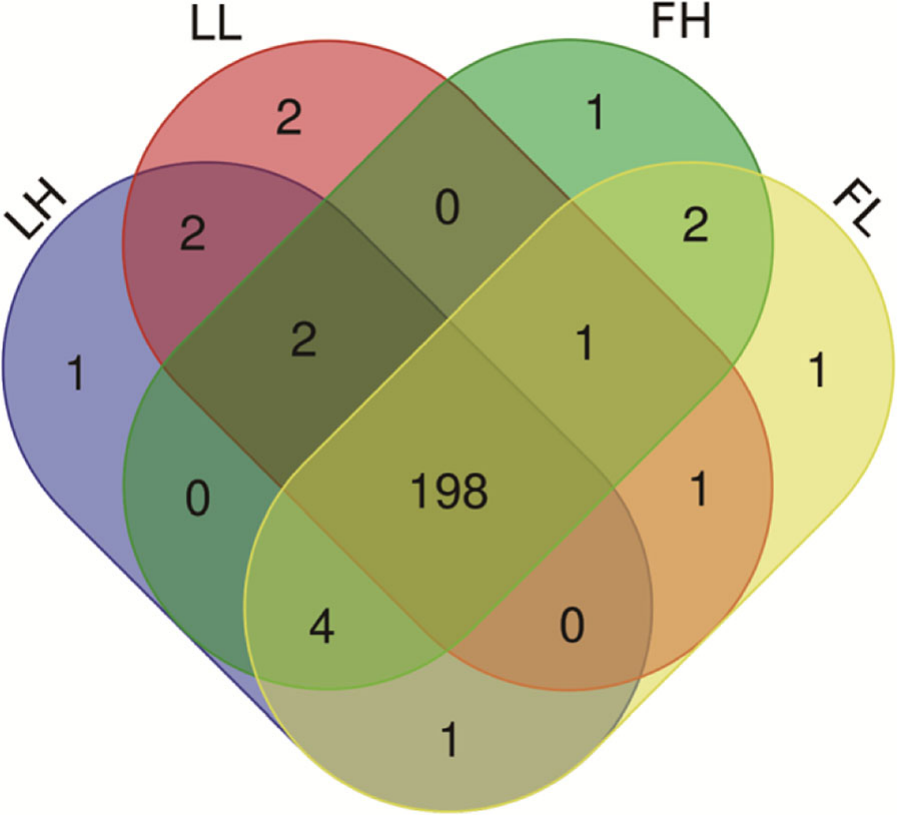
Venn diagrams of miRNAs. A total of 198 known miRNAs were shared in four groups (LH, LL, FH and FL)

Small RNA sequencing data from ovarian tissue from 16 sows was generated on Illumina HiSeq and provided a total of 433,792,963 raw reads. After filtering out the low-quality sequences, including adaptor dimmers and less than 18 nt, 363,273,750 clean reads were ultimately achieved. The percentage of clean reads ranged from 74.75 to 88.31% in each small RNA library (Additional file 4: Table S4–1). The length distribution of clean reads showed that most of the reads were 20–24 nt, and 22 nt was the most abundant length identified. Such reads accounted for 36.30% of the total sequences (Additional file 4: Table S4–2). In total, 216 known miRNAs (Additional file 5: Table S5–1) and 1724 novel miRNAs (Additional file 5: Table S5–2) were identified, while 198 known miRNAs were expressed in all four groups (Fig. 1).

### Genomic features and expression patterns of lncRNAs

**Fig. 2:**
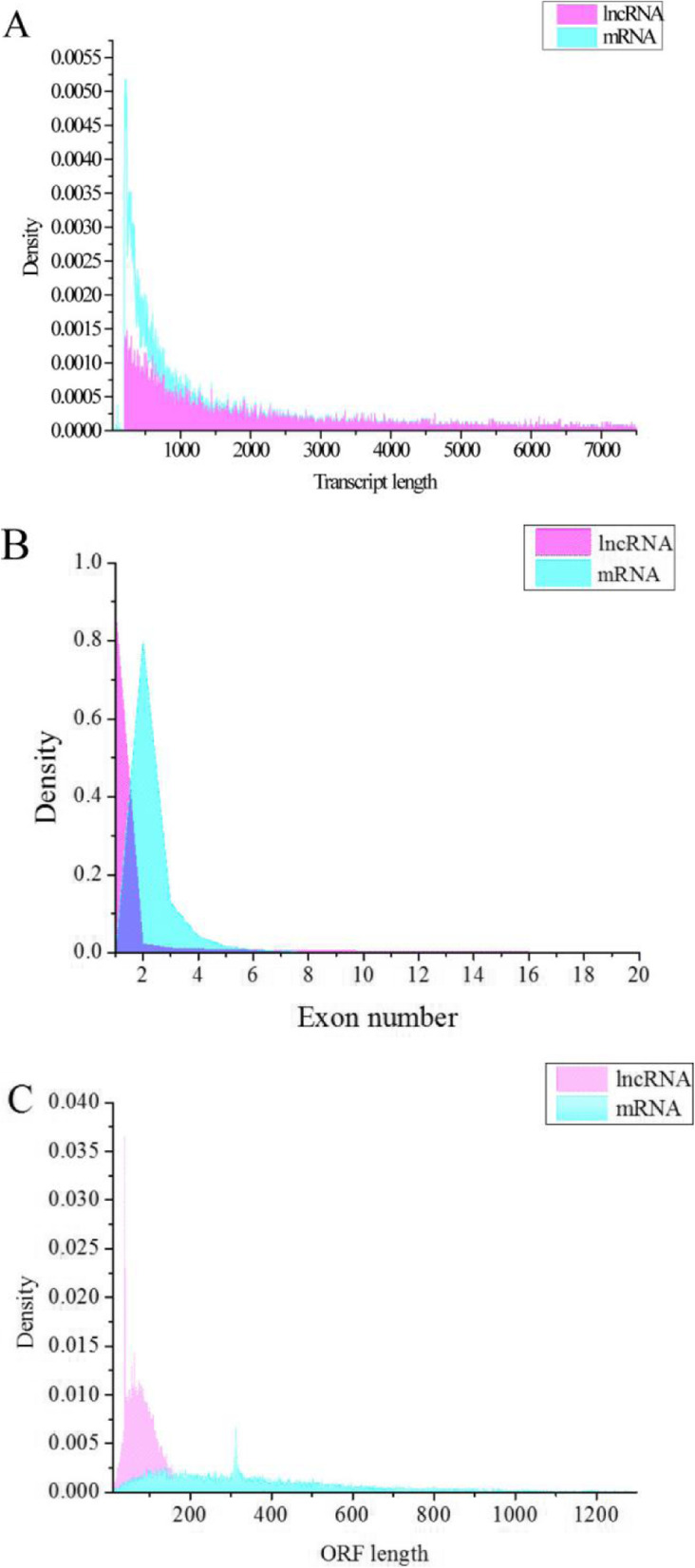
Genomic features of lncRNAs. **a** Length distribution of 27,370 mRNAs (pink) and24,447 lncRNAs (blue). **b** Exon number distribution of mRNAs and lncRNAs. **c** ORF length distribution of mRNAs and lncRNAs

**Fig. 3:**
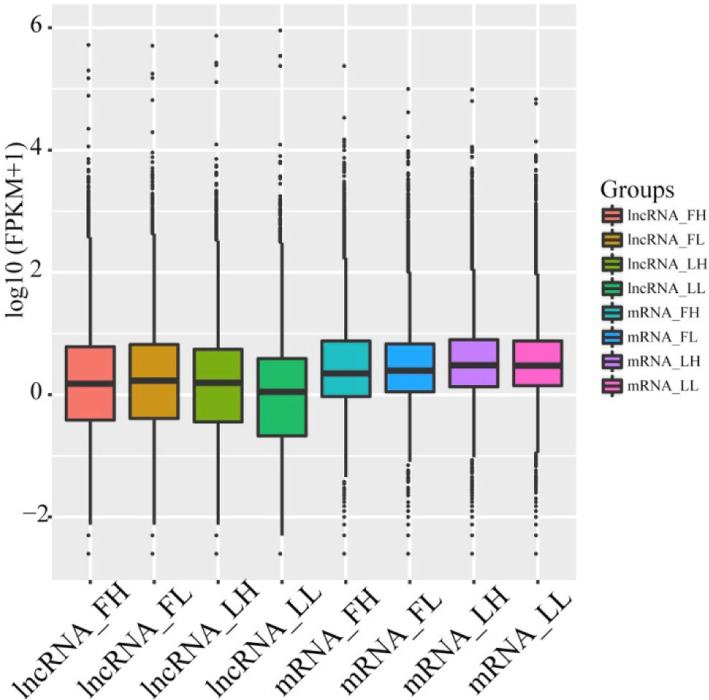
Expression levels of lncRNAs and mRNAs in four groups (LH, LL, FH and FL)

Overall, 24,447 lncRNAs and 27,370 mRNAs were detected in the ovaries of all 16 individual sows. In order to examine the differences in features between lncRNAs and mRNAs in ovarian tissues, their lengths were compared. The average length of lncRNAs was 2955 bp, which was longer than that of the mRNAs (Fig. 2a). We also observed that the number of exons of lncRNAs was lower than that of the mRNAs, which tend to contain 2.3 exons (Fig. 2b). The ORFs of the lncRNAs were shorter than those of the mRNAs (Fig. 2c). Lastly, their expression levels were also compared (Fig. 3); In general, lncRNAs had lower expression levels.

**Fig. 4:**
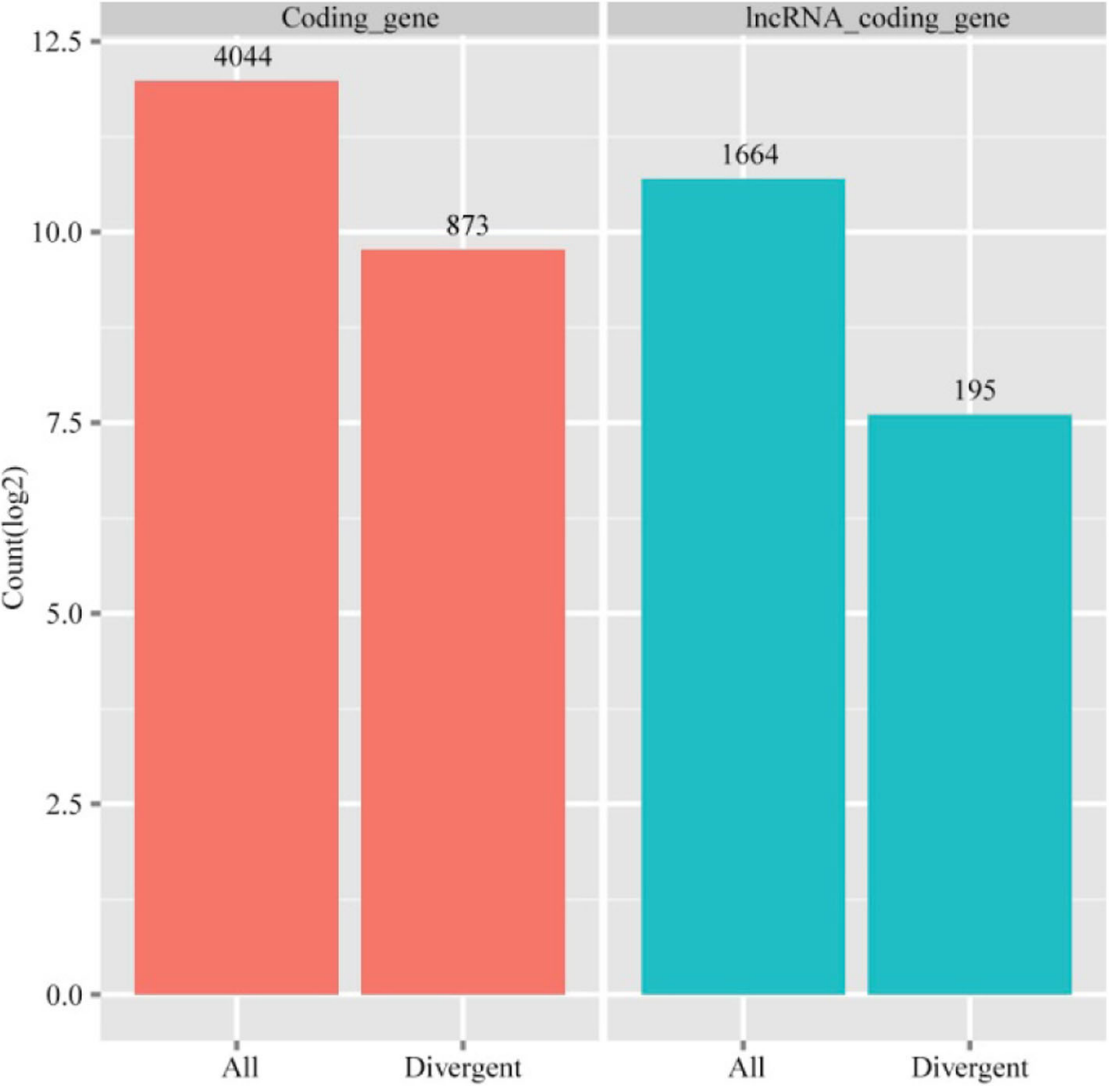
Number of gene pairs formed by lncRNAs and their neighboring coding genes. Proportion of divergent and all directions in coding gene pairs (red) and lncRNA: coding gene pairs (blue)

**Fig. 5:**
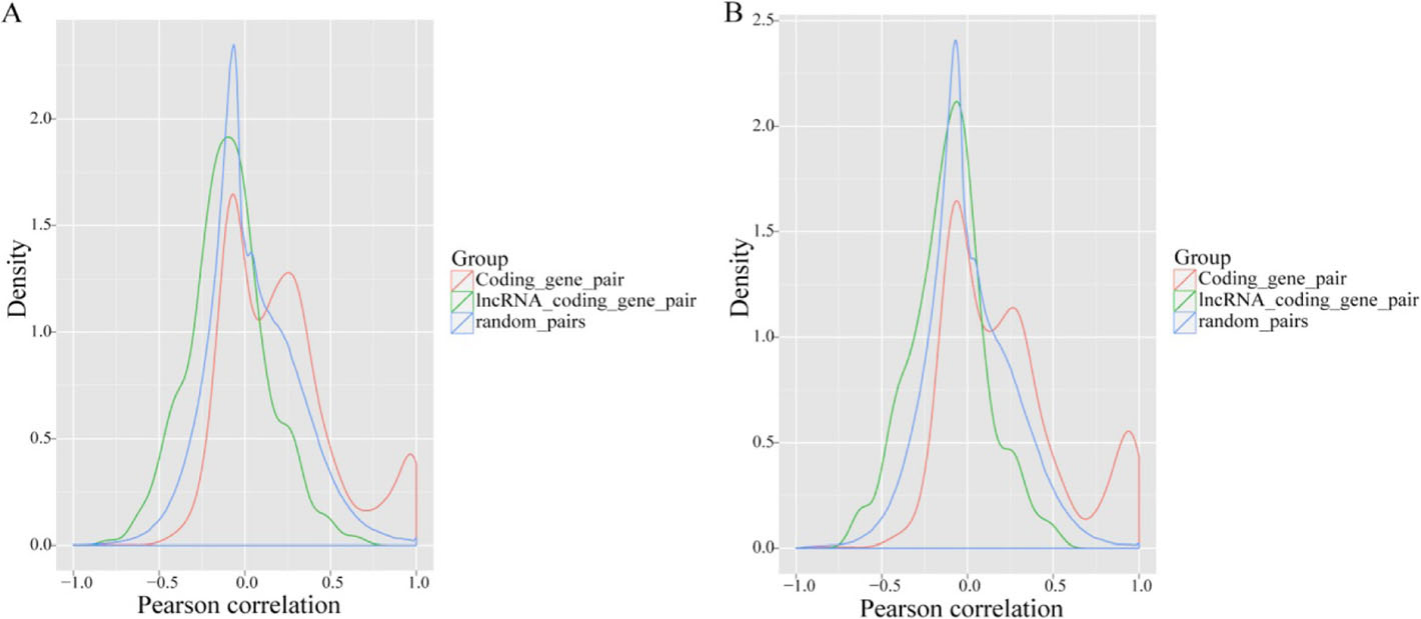
Correlation of expression patterns between pairs of neighboring genes. **a** distributions of Pearson correlation coefficients in expression levels between either 4004 pairs of coding gene neighbors (red), 1664 pairs of lncRNAs and their neighboring coding genes (green), or random pairs of genes (blue). **b** Distribution of Pearson correlation coefficients calculated as in A, but only for 195 pairs of divergently transcribed pairs of lncRNAs and protein-coding genes (green) or 873 pairs of divergently transcribed protein-coding genes (red)

lncRNAs can act in *cis* or *trans* to positively or negatively regulate gene expression; however, *cis*-acting lncRNAs are restricted to the chromosome from which they are transcribed [30]. Several studies also demonstrated that some lncRNAs have a high correlation with expression of neighboring gene [30, 31]. To further explore the relationship between lncRNAs and their neighboring coding genes in ovarian tissues, we searched for neighboring protein-coding genes (< 10 k) of all the identified lncRNAs and analyzed gene pairs formed by lncRNAs and their neighboring genes. We identified 4044 protein-coding genes: coding gene pairs (873 in divergent) and 1664 lncRNA: coding gene pairs (195 in divergent) (Fig. 4). We observed that the expression pattern of lncRNAs with their neighboring gene pairs (average Pearson correlation: 0.20) was similar to the coding gene pairs (average Pearson correlation: 0.28) and exhibited a significantly higher correlation than random coding gene pairs (average Pearson correlation: 0.041, *P* < 0.01) (Fig. 5a). We observed that there was a relatively low correlation between divergent lncRNAs: coding gene pairs (average correlation: 0.19) than divergent coding gene pairs (average Pearson correlation: 0.30, *P* < 0.05), and a higher correlation compared with random coding gene pairs (average Pearson correlation: 0.013, *P* < 0.01) (Fig. 5b). This result indicated that the correlation between lncRNAs and their neighboring gene was higher than random coding gene pairs.

### Identification of differentially expressed mRNAs, lncRNAs and miRNAs between the high and low fertility groups

From the expression profiles, differentially expressed mRNAs, lncRNAs and miRNAs in the ovaries of Large White pigs were obtained by comparing LH vs. LL and FH vs. FL (Table 1). A total of 956 (345 up-regulated and 611 down-regulated) lncRNA transcripts were differentially expressed in LH vs. LL (*P* < 0.05), while 415 (247 up-regulated and 168 down-regulated) were differentially expressed in FH vs. FL (*P* < 0.05) (Additional file 6: Table S6–1 and 2). We also identified 457 mRNA transcripts that were differentially expressed between the LH and LL groups (Table 1) (Additional file 6: Table S6–3). Among these transcripts, 334 were annotated as known genes. In the FH vs. FL comparison, we found that 475 mRNAs were differentially expressed, while 316 mRNAs were annotated (Additional file 6: Table S6–4). Analyses of the small RNA sequencing data showed that 29 and 11 known miRNAs were differentially expressed when comparing LH vs. LL and FH vs. FL, respectively (Additional file 6: Table S6–5 and 6).

**Table 1:** Number of differentially expressed genes in LH vs. LL and FH vs. FL

Genes	LH vs. LL		Total	FH vs. FL		Total
	Up- regulated	Down-regulated		Up-regulated	Down-regulated	
mRNAs	161	296	457	253	222	475
lncRNAs	345	611	956	247	168	415
miRNAs	68	54	122	32	14	46

### Function enrichment analysis of the lncRNAs

To investigate the function of the differentially expressed lncRNAs in each comparison, the potential targets of lncRNAs were predicted in this study. GO analysis revealed that there were 18 and 15 significant GO terms (corrected *P* < 0.05) in LH vs. LL and FH vs. FL, respectively (Additional file 7: Table S7–1 and 2). We noticed that three significant GO terms were common in all four groups: catalytic activity, single-organism metabolic process and vitamin D metabolic process. The KEGG analysis indicated that a total of 23 and 14 significant pathways were found in LH vs. LL and FH vs. FL, respectively (Additional file 7: Table S7–3 and 4). Importantly, it was observed that “ovarian steroidogenesis” and “lysosome” were the specific enrichment pathways in LH vs. LL, while steroid biosynthesis was the common pathway in the four comparison groups.

### Target prediction of miRNAs and construction of miRNA–mRNA networks

To understand the biological functions of differentially expressed miRNAs on fertility, we predicted the potential target genes of these miRNAs in two comparisons. We found that there were 13,458 putative target sites for 122 miRNAs in LH vs. LL and 4466 target sites for 46 miRNAs in FH vs. FL (Additional file 8: Table S8–1 and 2). Furthermore, GO and pathway enrichment analyses were performed. GO analysis of the target genes revealed that there were 410 and 236 significant GO terms (corrected *P* < 0.05) in LH vs. LL and FH vs. FL comparisons (Additional file 9: Table S9–1 and 2). KEGG pathway analysis revealed that a total of 97 and 31 significant pathways (Hypergeometric Distribution Hypothesis Test, *P* < 0.05) were identified in LH vs. LL and FH vs. FL comparisons, respectively (Additional file 9: Table S9–3 and 4). Among these KEGG pathways, multiple pathways were closely involved in the reproductive process, such as the Insulin signaling pathway, MAPK signaling pathway, Estrogen signaling pathway, GnRH signaling pathway, PI3K-Akt signaling pathway, Ras signaling pathway, Cytokine-cytokine receptor interaction, Jak-STAT signaling pathway and Lysosome pathway in LH vs. LL, and the Notch signaling pathway, TGF-beta signaling pathway and Steroid biosynthesis in FH vs. FL. It is worth noting that the Wnt signaling pathway, Insulin secretion and Adherens junction were common in LH vs. LL and FH vs. FL.

**Fig. 6:**
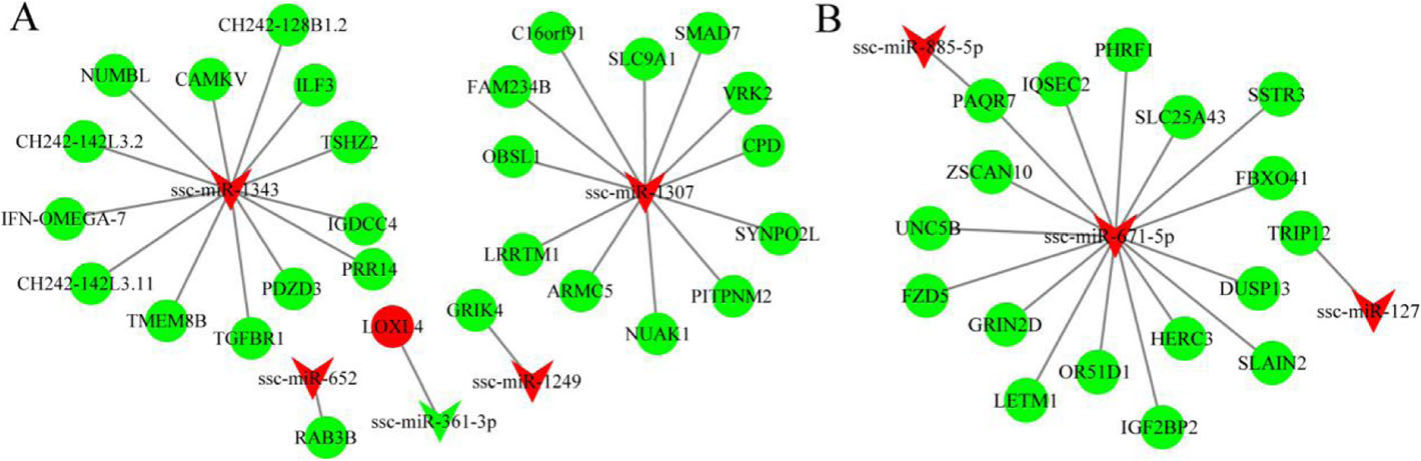
miRNA–mRNAs interaction network constructed and visualized. VEE and circular nodes represent miRNAs and mRNAs, respectively. Red nodes represent up-regulation, while green nodes represent down-regulation. **a** Interaction networks for comparison of LH vs. LL; **b** Interaction networks for comparison of FH vs. FL

Additionally, we aimed to illustrate negative interactions between differentially expressed miRNAs and mRNAs in the porcine ovary that might lead to differences in fertility; thus, regulatory networks of miRNA–mRNA pairs were constructed (Fig. 6). Of these negative interactions, three miRNAs (ssc-miR-1307, ssc-miR-1343 and ssc-miR-671-5p) targeted multiple mRNAs, but several miRNAs targeted only one mRNA. For example, up-regulated ssc-miR-1307 targets 12 genes, but down-regulated ssc-miR-361-3p targets only one gene (Fig. 6a). Moreover, down-regulated progestin and adipoQ receptor 7 (PAQR7) (FH vs. FL) was regulated by two differentially expressed miRNAs including ssc-miR-885-5p and ssc-miR-671-5p (Fig. 6b).

### Construction of lncRNA-miRNA-mRNA networks

**Fig. 7:**
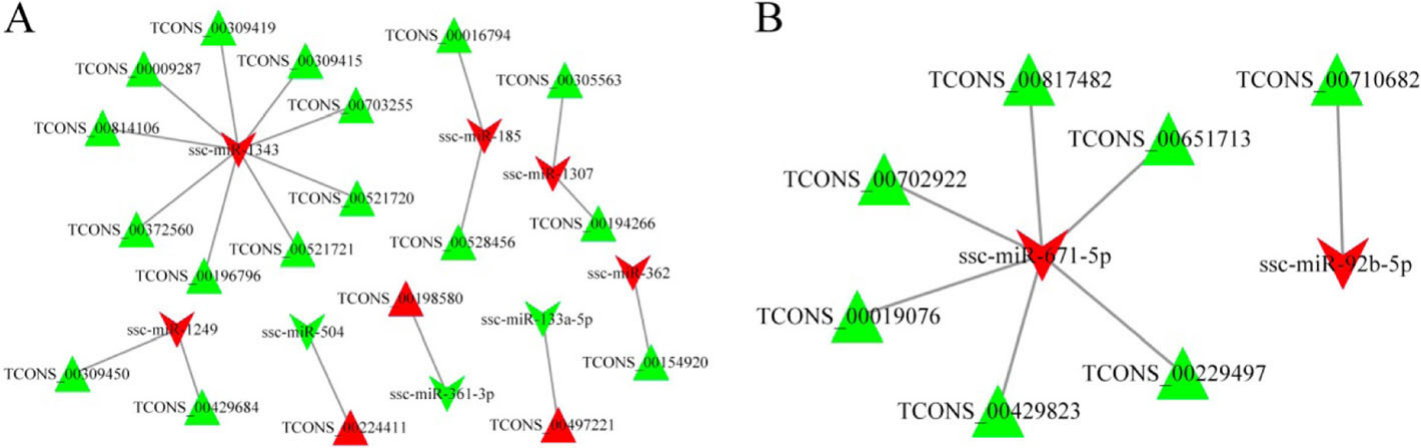
Interaction networks of differentially expressed lncRNAs and miRNAs in ovarian tissue. Triangular nodes denote lncRNAs and VEE nodes denote miRNAs. Red nodes represent up-regulation and green nodes represent down-regulation. **a** Interaction networks for comparison of LH vs. LL. **b** Interaction networks for comparison of FH vs. FL

To explore the role and relation of lncRNAs and miRNAs mediation in pig fertility, differentially expressed lncRNAs were selected by miRanda analysis [32]. The lncRNA–miRNA negative pairs between differently expressed lncRNAs and miRNAs were selected to construct the co-expression network. In the LH vs. LL comparison, we found that the key miRNAs interacted with 19 lncRNAs (Fig. 7a). In FH vs. FL group, the key miRNAs interacted with 7 lncRNAs (Fig. 7b). A total of 19 and 7 lncRNA–miRNA pairs were identified in LH vs. LL and FH vs. FL, respectively. It is worth noting that most lncRNAs were targeted by the same miRNA. Among these key miRNAs, ssc-miR-1343 and ssc-miR-671-5p had more interactions than others. Ssc-miR-1343 is the key miRNAs targeted with nine key lncRNAs (TCONS_00009287, TCONS_00196796, TCONS_00309415, TCONS_00309419, TCONS_00372560, TCONS_00521720, TCONS_00521721, TCONS_00703255, and TCONS_00814106) through MREs, and ssc-miR-671-5p targeted with six key lncRNAs (TCONS_00019076, TCONS_00229497, TCONS_00429823, TCONS_00651713, TCONS_00702922, and TCONS_00817482), which may be key regulators related to fertility.

**Fig. 8:**
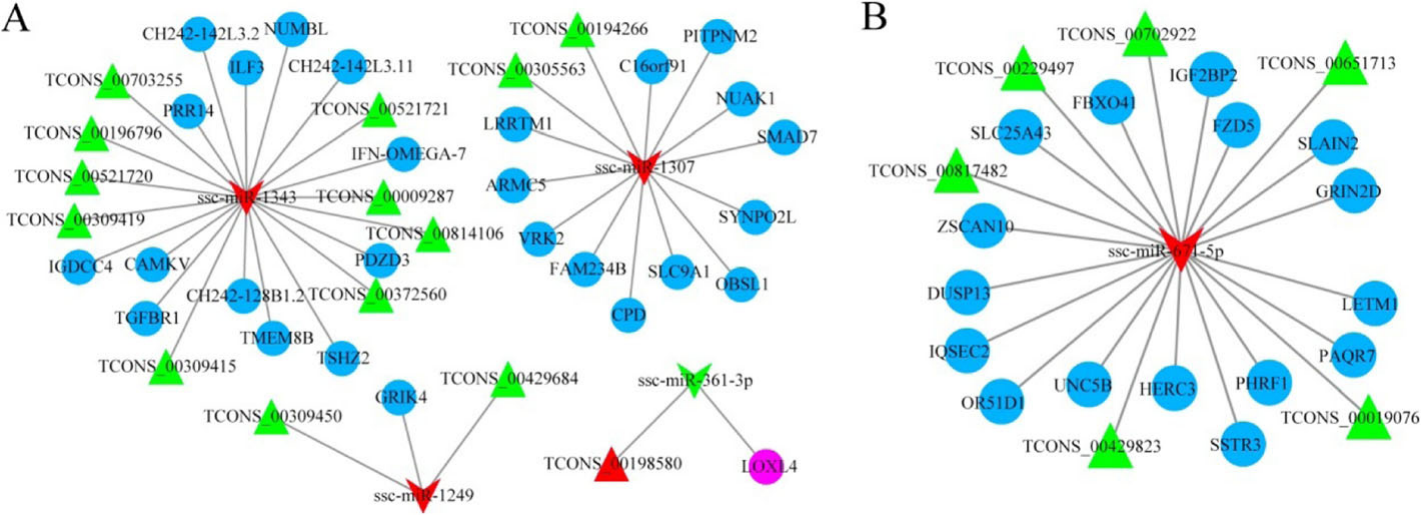
lncRNA–miRNA–mRNAs interaction network constructed and visualized. Red triangle nodes represent up-regulated lncRNAs, and green nodes represent down-regulated lncRNAs. Red VEE nodes indicate up-regulated miRNAs, and green nodes indicate down-regulated miRNAs. Purple circular nodes represent up-regulated mRNAs, and blue represent down-regulated mRNAs. **a** Interaction networks for comparison of LH vs. LL; **b** Interaction networks for comparison of FH vs. FL

Based on the above data, we integrated the lncRNA–miRNA interactions and miRNA–mRNA interactions to establish lncRNA–miRNA–mRNA networks and then visualized using the Cytoscape software (Fig. 8). The network of LH vs. LL was composed of 44 nodes and 40 edges, and the nodes included 4 miRNAs, 14 lncRNAs and 27 mRNAs, which could be the important nodes involved in the ceRNA network during the luteal phase of the estrous cycle (Fig. 8a). In this network, some of them have been reported to be reproduction-associated molecules such as *NUMBL*, *ILF3*, *GRIK4*, *SLC9A1, TGFBR1*and *LOXL4*. We noticed that nine lncRNAs were interrelated with ssc-miR-1343 and may act as ceRNA to inhibit target miRNAs and mediated, related hub genes translation such as *NUMBL*, *ILF3*, *TGFBR1*, *TMEM8B*, *PRR14*, *TSHZ2*, and *CAMKV*. In addition, we found that TCONS_00309450 and TCONS_00429684 may serve as ceRNA to mediate *GRIK4* by sponging ssc-miR-1249. In the FH vs. FL group, there were 23 nodes and 22 edges, consisting of 1 miRNA, 6 lncRNAs and 16 mRNAs (Fig. 8b). These six lncRNAs may serve as ceRNA to mediate the corresponding gene transcripts by sponging ssc-miR-671-5p. We also found that several genes, such as *GRIN2D* and *FZD5,* were mainly involved in the “cAMP signaling pathway”, “Calcium signaling pathway”, “Wnt signaling pathway” and “mTOR signaling pathway”, implying that they might be acting as reproduction related genes. Thus, we hypothesize that these lncRNAs, miRNAs and mRNAs play critical roles in fertility regulation.

### RT-qPCR verification

**Fig. 9:**
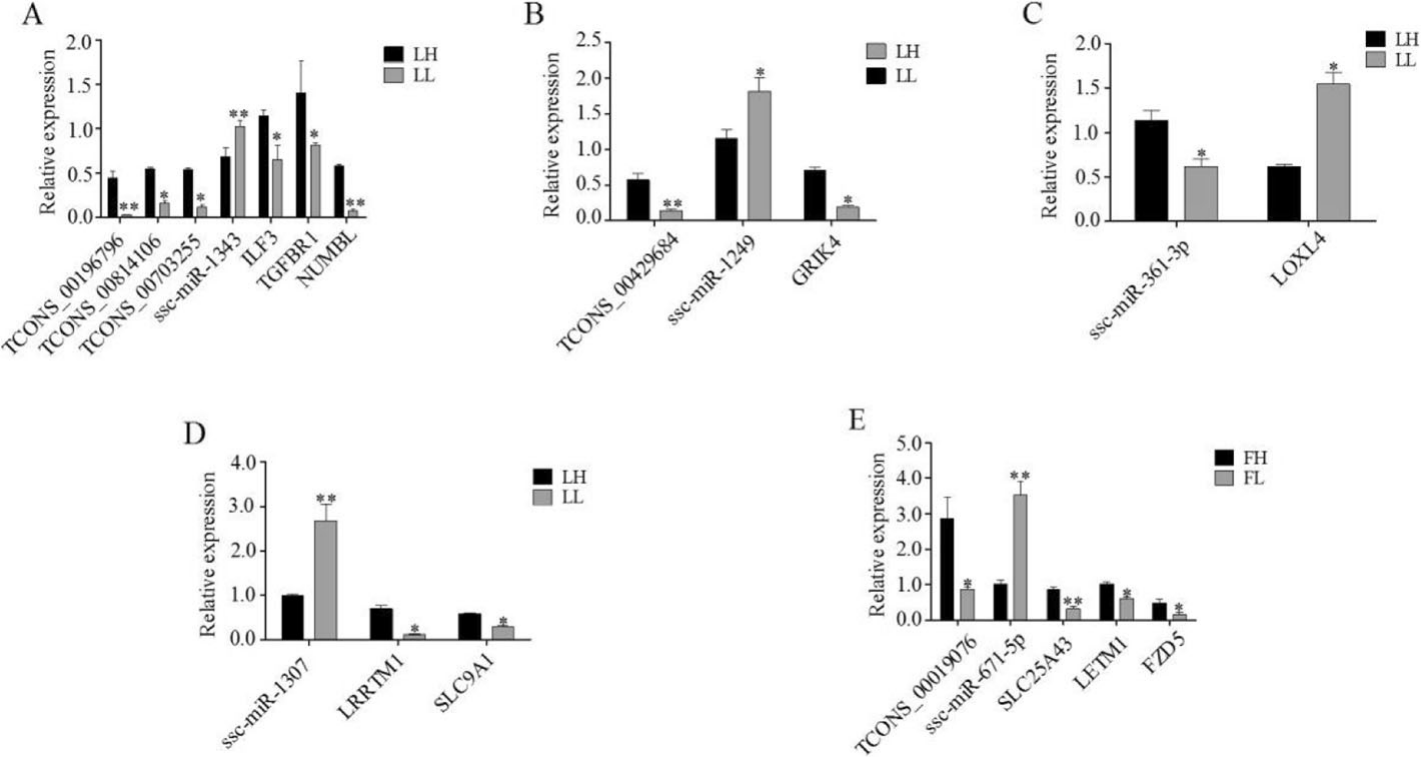
Expression levels of key genes by RT-qPCR. **a-d** LH vs. LL. **e** FH vs. FL. Data are presented as the mean ± SD of three experiments. The data statistical significance was assessed by Student’s *t*-tests. *P ≤ 0.05, **P ≤ 0.01 (Compared with the high fertility)

In the lncRNA–miRNA–mRNAs interaction networks, we selected 14 and 5 key nodes to validate expression levels in LH vs. LL and FH vs. FL, respectively, using RT-qPCR (Fig. 9). The expression of each miRNA was significantly higher in the LL groups compared to the LH groups. In contrast, the expression of each lncRNA or mRNA was significantly lower in the LL groups than in the LH groups (Fig. 9a-d). We also found that the expression of ssc-miR-671-5p was higher in the FL groups compared to the FH groups (Fig. 9e). These results suggested that the post-transcriptional regulatory functions of miRNAs negatively correlated with their targets and that these differentially expressed miRNAs and lncRNAs may contribute to the fertility differences in sows with extreme phenotypes during the F and L phases of the estrous cycle. In brief, the results demonstrated that the expression patterns of 19 differentially expressed genes were consistent between the RT-qPCR data and the RNA-Seq data, implying that the accuracy of RNA-Seq data was reliable (Fig. 9).

## Discussion

Fecundity is of primary interest in pigs as it plays a critical role in efficiency of production. Litter size, such as TNB, is one of the most important reproductive traits that are difficult to improve by selection because of its low heritability [1]. Meanwhile, it is a highly complex trait, which is controlled by multiple genes and is affected by parity, environmental factors and sire [3–6]. More importantly, ovulation rate is also a major factor that affects the litter size of sows [33]. Several studies in the pig and goat have reported some fecundity-related candidate genes based on RNA-seq data from the ovarian tissues with high or low littler size [23, 34]. Although a previous report described miRNA expression profiles in pig ovarian tissues correlating with fecundity [25], much less is known about the regulatory molecular mechanisms of fertility in sows. In addition to miRNAs, lncRNAs have been found to play critical roles in transcriptional and post-transcriptional regulation [19]. However, no reports to-date have focused on lncRNA functions in pig fertility. In this study, a total of 16 sows were selected according to the methods in previous studies [25, 34]. Eight sows from H fertility groups with high TNB per birth; similarly, eight sows from L fertility groups with low TNB per birth. Meanwhile, in order to minimize the effects of parity on TNB, eight sows with similarly high or low parity from each group were chosen for the study. Due to the dynamic nature of the mammalian ovary, we selected ovarian tissues at the follicular and luteal phase to study the differences between the high and low fertility in Large White sows using highthroughput sequencing technology. We systematically analyzed the expression of lncRNAs in porcine ovaries, and comprehensively integrated mRNA and miRNA data to identify the lncRNA-miRNA-mRNA interactions mediated in the competing endogenous RNA (ceRNA) network to further elucidate the regulatory mechanisms of lncRNAs in sow fertility.

lncRNAs expression profiles in ovarian tissues at different stages of follicle development in pigs were first investigated using RNA sequencing analysis by Liu et al. (2018) [28]. In the present study, we used the pig genome assembly (Sscrofa 10.2) to analyze the expression profiles of each sample, which is consistent with other published studies [28–30, 35, 36]. Based on comparative analysis, we found that the number of lncRNAs (*n* = 24,447) detected in this study was much higher than that reported in the study by Liu et al. (*n* = 2076) [28]; however, the number was much lower compared to that of human lnRNAs reported in LNCipedia 4.1 [37]. We also observed that the lncRNAs identified in ovarian tissues have fewer exons, lower expression levels, and shorter ORFs than those of the protein-coding genes (Fig. 2b-c and Fig. 3). Our results are consistent with previous studies of thyroid glands and endometriums of pigs [30, 35]. However, our results also showed that the average length of lncRNAs in ovarian tissues were longer than that in the thyroid gland (2337 bp on average) [35], fetal skeletal muscle (1043 bp on average) [36] and endometrium of pigs (1454 bp on average) [30]. Although there are some improvements in current pig genome assembly (Sscrofa11.1) compared to the Sscrofa10.2, a recent study based on Isoform Sequencing (Iso-Seq) data sourced from nine porcine tissues have revealed that the current pig genome annotations are still incomplete [13]. However, in this study, using RNA-seq data from 16 porcine ovary samples, we could discovered 24,447 lncRNAs that is similar to Beiki et al. (2019) predicted lncRNA transcripts (*n* = 24,527) [13].

As an important post-transcriptional regulatory factor, miRNAs play an essential role in diverse biological processes. In the present study, ssc-miRNA-26a and ssc-miRNA-99a were differentially expressed between the high and low fertility groups in LH vs. LL (Additional file 6: Table S6), which is in accordance with the findings of Huang et al. (2016) [25]. A previous study reported that miR-26a was significantly up-regulated in chicken ovarian follicles and is likely to be associated with the mechanism of recruitment of dominant follicles [38]. We therefore hypothesize that the two miRNAs may have important roles in reproductive physiology.

Accumulating evidence has demonstrated that miRNAs are important endogenous regulators of gene expression, which have been investigated in various biological mechanisms [24, 39–41]. However, with the increasing amounts of discovered lncRNAs, the function of very few lncRNAs has been characterized. Recent studies have demonstrated that lncRNAs can act as endogenous miRNA sponges, thereby reducing the negative regulatory effect of miRNAs on mRNAs [42]. Although the ceRNA networks are receiving research attention, most of the relevant studies have focused on their effects related to human diseases.

Apart from miRNAs, lncRNAs in the developing ovary have also been implicated in improving fecundity [23]. However, the potential roles of lncRNAs in regulation of porcine fertility are far from understood. Miao et al. (2016) constructed a miRNA–lncRNA–mRNA interaction network based on the competing endogenous RNA (ceRNA) hypothesis, which provided a new insight into understanding sheep fertility. In the present study, we identified differentially expressed lncRNAs, miRNAs and mRNAs between the high and low fecundity groups in ovarian tissues. Then, we systematically analyzed the complex effects of the interactions between miRNAs and their target genes in LH vs. LL and FH vs. FL groups. Lastly, we constructed new ceRNA networks to comprehensively investigate the potential relationships between lncRNAs and miRNAs in sow fertility.

By constructing lncRNA–miRNA–mRNA regulatory networks using bioinformatics, we identified three miRNAs, ssc-miR-1249, ssc-miR-1307 and ssc-miR-1343, which exhibited significant up-regulation in the LL group compared to the LH group (Fig. 8a). The reliability of their expression patterns was confirmed by RT-qPCR (Fig. 9a, b and d). Among these miRNAs, miR-1249 has been reported to promote the proliferation of glioma cells [43]. Previous work in bull spermatozoa has shown that miR-1249 correlates with fertility rates [44]. The expression of miR-1249 was found to be significantly higher in bulls with moderate fertility compared with the high-fertility group, indicating that miR-1249 negatively regulates the expression of protein-coding genes, which leads to problems during reproduction [44]. Remarkably, miR-1249 was located on *BTA5,* which has been a candidate gene associated with reproduction efficiency in cattle [45]. These results further suggested that miR-1249 might plays important roles infertility regulation. In this network, we determined that the TCONS_00429684/TCONS_00309450–ssc-miR-1249–GRIK4 interaction axis was involved in the regulatory network. Concerning mRNAs, function enrichment analyses showed that *GRIK4* has been involved in a reproduction-relevant pathway, such as neuroactive ligand-receptor interactions, which play important roles in reproduction processes [46]. In the present study, high levels of *GRIK4* expression was noticed in the LH group compared with the LL group. Meanwhile, the RT-qPCR results also revealed that the expression pattern of *GRIK*4 was consistent with TCONS_0042968. However, the expression of ssc-miR-1249 was significantly increased in the LL group, which means that it might inhibit the transcription of *GRIK4* and exert a negative impact on the fertility of pigs. Thus, we speculate that the high expression of lncRNAs (TCONS_00429684 and TCONS_00309450) in the LH group may be through the absorption of ssc-miR-1249 to promote *GRIK*4 transcription. Despite our observations, the underlying mechanisms need further investigation.

We also found that ssc-miR-1343 and ssc-miR-1307 played central roles in the regulation network (Fig. 8a). No previous study has reported the expression of ssc-miR-1307 and ssc-miR-1343 in ovarian tissue. According to our results, ssc-miR-1307 and ssc-miR-1343 showed significant up-regulation in the LL group. The RT-qPCR results revealed that the expression patterns of Interleukin Enhancer Binding Factor 3 (ILF3), Numblike (NUMBL) and Transforming Growth Factor-β Receptor Type I (TGFBR1) were consistent with several lncRNAs (Fig. 8a). Some studies showed that *ILF3* is strongly expressed in the mouse ovary and that the ILF3 protein predominantly functions in germ cells [47]. The *ILF3* protein was frequently detected in adult zebra fish gonads by global proteomics [48], and may be involved in gonad differentiation [49]. In addition, the Notch signaling pathway could be involved in the development of organs and tissues by regulating cellular developmental processes, such as cell proliferation, differentiation and apoptosis [50]. A recent publication by Jing et al. (2017) reported that the Notch signaling pathway could promote ovarian follicular development by regulating the growth and estradiol production of granulosa cells [51]. *NUMBL*, a known antagonist of Notch signaling, has been implicated in gonadal development. Research has confirmed that deletion of *NUMBL* disrupted coelomic epithelium cell polarity in both XX and XY gonads, and germ cell numbers were also reduced at early stages of gonadogenesis, suggesting a major role in gonad development [52]. Another key gene, *TGFBR1*, is known to be a type I receptor of the canonical TGF-β signaling pathway. Increasing evidence has shown that *TGFBR1* is closely related to ovulation rate and litter size [53, 54]. In this study, ssc-miR-1343 had the most interactions in the network, which is a bridge of *ILF3*, *NUMBL*, *TGFBR1* and several lncRNAs. Based on the combined detection of their expression, we speculate that these lncRNAs might be associated with reproductive efficiency in sows.

In addition to FH vs. FL, we analyzed the relationship among lncRNAs, miRNAs, and mRNAs in ovarian tissues, as shown in Fig. 8b. We found that miR-671-5p had the most interactions, indicating that it is the hub gene in the regulation network. We further observed that miR-671-5p potential target gene *FZD*5 was largely involved in the Wnt and mTOR signaling pathways, which plays a critical role during the estrous cycle [55, 56]. Specifically, the TCONS_00019076–miR-671-5p–FZD5 interaction is involved in the regulatory network. The RT-qPCR results revealed that the expression of *FZD*5 was significantly up-regulated in the FH groups, which was consistent with the results from the RNA-seq data. Furthermore, we showed that the level of TCONS_00019076 was up-regulated in the LH groups, which was consistent with the expression pattern of *FZD*5. Given the potential binding sites between TCONS_00019076– miR-671-5p and miR-671-5p–FZD5, we propose that TCONS_00019076 may promote the expression of *FZD*5 through the absorption of miR-671-5p. In addition, two of the 16 mRNAs, *PAQR*7 and *IGF*2*BP*2, have been reported to be related to oocyte maturation and cell proliferation [57, 58]. Thus, we suggest that these key lncRNAs may play an important role in the regulation of pig fertility. In future research, we plan to explore the function of these lncRNAs using overexpression and knockdown experiments.

## Conclusions

In this study, ovarian lncRNAs and miRNAs associated with prolificacy of Large White sows were identified during the follicular and luteal phases of the estrous cycle, and their potential biological functions were predicted through bioinformatics. In addition, we constructed interaction networks among a series of differentially expressed lncRNAs, miRNAs, and mRNAs in ovarian tissues using an integrative biology approach. Our data will be helpful for identifying a novel regulatory mechanism for investigating prolificacy in pigs in future studies.

## Methods

### Swine population pool, experimental design and tissue collection

All of the experiments involving animals were carried out in accordance with the guidelines for the care and use of experimental animals established by the Ministry of Science and Technology of the People’s Republic of China (Approval Number 2006–398), and the work was approved by the Animal Ethics Committee at Hebei Agricultural University, China.

The animals (sows, *n* = 590; boars, *n* = 42) used in this study, a Canadian Large White population, were from the Hebei Shunde-Tianzhao Livestock Technology Co., Ltd. (Wanquan, Hebei, China). Detailed documents of 590 multiparous sows were collected, and the total number of piglets born (TNB) was regarded as an important parameter to evaluate the fertility of the animals as previously described [25, 34, 59, 60]. For this study, the data of TNB obey the normal distribution. Which was calculated using the SPSS19.0 software package (IBM Corp, Armonk, NY, USA). The high (H; TNB > 15.73) and low (L; TNB < 11.11) fertility groups of sows were determined according to our previous study [59]. Eight sows with similarly high or low parity from each group were chosen for the study (*n* = 8 for the H group and *n* = 8 for the L group). The sows were reared under the same environmental conditions and allowed ad libitum access to feed and water, and they were individuals without kinship according to the traceability system. All phenotypic records are displayed in Additional file 10: Table S10. Sows with H and L fertility were sacrificed at each of the two stages: on day 14 (day 1 = first day of estrus) after estrus in the luteal (L) phase (LH: *n* = 4; LL: *n* = 4) and on day 20 after estrus in the follicular (F) phase (FH: *n* = 4; FL: *n* = 4) [61]. At the above time points, animals were humanely slaughtered by electronic stunning followed by exsanguinations, and the ovarian tissues were rapidly removed and snap-frozen in liquid nitrogen until subsequent processing.

### Library preparation and solexa sequencing

Total RNA was extracted from each ovarian sample, and then purified with RNeasy Mini Kit (Qiagen, Valencia, CA, USA). Total RNA from each sample was quantified and qualified as previously described [59]. The same sample was used for both sequencing and RT-qRCR analysis.

Approximately 3 μg of RNA of each RNA sample was used for library preparations. To remove ribosomal RNA (rRNA), the Epicentre Ribo-Zero™ rRNA removal Kit (Epicentre, Madison, WI, USA) was used. Then, the rRNA-depleted RNA was used to generate cDNA libraries using the NEB Next® Ultra™ The Directional RNA Library Prep Kit for Illumina® (New England Biolabs; NEB, Ipswich, MA, USA) according to the manufacturer’s protocol. Sequencing was carried out using a 2 × 150 paired-end (PE) configuration on Illumina HiSeq X10 (Illumina, San Diego, CA, USA).

For small RNA sequencing, the same samples were used to construct Illumina small RNA-Seq (RNA sequencing) libraries using the NEBNext® Multiplex Small RNA library Prep Set for Illumina® (NEB) following the manufacturer’s recommendations. In brief, 3′ SR Adaptor for Illumina was ligated to the small RNA using 3′ Ligation Enzyme. The 5′ SR Adaptor for Illumina was ligated to the small RNA using 5′ Ligation Enzyme and first strand cDNA was synthesized using ProtoScript II Reverse Transcriptase (NEB). Each sample was then amplified by PCR for 12 cycles using P5 and P7 primers, with both primers carrying sequences which can anneal with flow cell to perform bridge PCR and P7 primers carrying a six-base index, thus allowing multiplexing. The PCR products of ~ 140 bp were recovered and cleaned up using PAGE. Purified small RNA sequencing libraries were validated using an Agilent 2100 Bioanalyzer (Agilent Technologies), and quantified by a Qubit 2.0 Fluorometer (Invitrogen). Finally, the 16 small RNA libraries were sequenced using a 1 × 50 single-end (SE) sequencing strategy by Illumina HiSeq X10 (Illumina).

### Analysis of RNA-Seq data

Raw RNA-seq reads from each sample were first processed by removing adapters and low-quality reads. Clean data from each library were obtained and then mapped to the Sscrofa10.2 reference genome that was downloaded from the Ensembl Genomes (ftp://ftp.ensembl.org/pub/release-86/fasta/sus_scrofa/dna/Sus_scrofa.Sscrofa10.2.dna.toplevel.fa.gz). The mapped reads from each individual animal were assembled as previously described [59]. Rsem (V1.2.6) [62] was used to calculate gene expression levels using the FPKM (Fragments per Kilo bases per Million reads) method for both the lncRNAs and coding genes in each sample. Additionally, the method of differential gene expression analysis was described in detail in ref. [59]. Transcripts of genes showing *P* values < 0.05 and with a |log_2_ (fold change)| ≥ 1 were identified as differentially expressed. The RNA-Seq data is publicly available in the NCBI GEO database with the accession number GSE134001.

### Analysis of microRNA-Seq data (miRNA)

Raw reads from the 16 small RNA libraries were generated. Clean reads were obtained by masking adapters, poly-A tails and low-quality reads from the raw data with Trimmomatic10 (v0.30) [63]. The clean reads were then mapped to the Sscrofa10.2 reference genome (ftp://ftp.ensembl.org/pub/release-86/fasta/sus_scrofa/dna/Sus_scrofa.Sscrofa10.2.dna.toplevel.fa.gz) by Bowtie 2 (v2.1.0) [64]. Subsequently, unmapped reads were used to predict the novel miRNAs with miRDeep (v2.0.0.7) [65]. Differential expression analysis was performed using a procedure described previously [60]. *P* values of miRNAs were settled at < 0.05 and |log_2_ (fold change)| ≥ 1 were described to detect differentially expressed miRNAs. The microRNA-Seq data is publicly available in the NCBI GEO database with the accession number GSE132307.

### Target prediction of miRNAs and construction of miRNA–mRNA networks

To explore the function of the miRNA, potential target genes of miRNAs with differential abundances were predicted by miRanda [32]. Subsequently we utilized GO-TermFinder (v0.86) (https://metacpan.org/release/GO-TermFinder) to identify Gene Ontology (GO) terms that annotated a list of enriched genes with a significant *P* value less than 0.05. The enrichment of KEGG pathways was tested using in-house scripts. In order to explore the potential interactions of miRNA and mRNA, miRNA–mRNA negative interactions were predicted, and Cytoscape_v3.5.1 [66] was used to construct the important networks of differentially expressed mRNAs and miRNAs.

### lncRNAs identification

According to the characteristics of the lncRNA, all the assembled transcripts were merged and then filtered by known non-lincRNA annotation, non-lincRNA characters, open reading frames (ORFs) and protein-coding potential methods. Known non-lincRNA include known protein-coding RNA, miRNA, tRNA, snoRNA, rRNA and pseudogenes. The characters of non-lincRNA include transcripts with more than one exon and with a length > 200 bp. For protein-coding potential prediction, we used CPC (Coding Potential Calculator) [67], CNCI (coding-noncoding-index) [68], CPAT (coding potential assessment tool) [69] and Pfam-scan [70] to distinguish mRNAs from lncRNAs. To understand the differences between lncRNAs and mRNAs, the genomic features of the predicted lncRNAs were analyzed [71].

### LncRNAs target gene prediction and GO and KEGG enrichment analyses

Transcriptional regulation by lncRNAs can work either in *cis* or in *trans* and may negatively or positively control gene expression [72]. As a result, the prediction of lncRNA target genes in *cis* and *trans* forms were performed. We searched coding genes 10 k upstream and downstream regions of lncRNAs as the *cis* target gene. Regulation in *trans* was analyzed by expression levels, according to Pearson’s correlation coefficient (|r| > 0.95).

To investigate the function of differentially expressed lncRNAs, GO enrichment analysis was implemented using GO-TermFinder (v0.86), and corrected *P* values < 0.05 were treated as significantly enriched. KEGG pathway analysis was performed using in-house scripts.

### Construction of lncRNA–miRNA–mRNA networks

To infer the function of lncRNAs, differently expressed lncRNAs were selected, and then lncRNA–miRNA negative interactions were predicted using miRanda [32]. Subsequently, based on complementary pairs between miRNA and mRNAs and between miRNAs and lncRNAs, the lncRNA–miRNA–mRNA interaction networks were constructed and visualized by Cytoscape_v3.5.1 [66].

### Reverse transcription real-time quantitative PCR (RT-qPCR)

Based on the lncRNA–miRNA–mRNA correlation networks, specifically, several interaction nodes were validated by RT-qPCR. For mRNA and lncRNA, reverse transcription of total RNA was performed using a cDNA Synthesis Kit (Sinogene, Beijing, China). For miRNA, total RNA was reverse-transcribed into cDNA using a One-Step miRNA RT Kit (Sinogene) following the manufacturer’s instructions. RT-qPCR was carried out using StepOne real-time PCR systems (Applied Biosystems, Foster City, CA, USA) and SYBR Green qPCR Mix (Sinogene). All amplifications were followed by dissociation curve analysis of the amplified products. All primer sequences, including selected genes, miRNAs and internal control genes (ACTB and U6 snRNA), are displayed in Additional file 11: Table S11. Relative expression levels of genes and miRNAs were calculated by the2^−ΔΔCt^ method [73].

### Statistical analysis

Statistical analyses were performed using SPSS 19.0 statistical software (IBM Corp). The data are expressed as the mean ± standard deviation (SD) of three independent experiments. When comparisons were made, Student’s *t*-tests were performed, and *P* < 0.05 was considered as statistically significant.

## Supplementary information

**Additional file 1: Table S1.** Summary of the sequencing reads alignment to the reference genome.

**Additional file 2: Table S2.** Identification of lncRNA transcripts of 16 libraries by RNA-sequencing.

**Additional file 3: Table S3.** Summary of mRNA transcripts of 16 libraries by RNA-sequencing.

**Additional file 4: Table S4.** Summary of draft reads of 16 libraries by small RNA-sequencing.

**Additional file 5: Table S5.** Information of miRNAs.

**Additional file 6: Table S6.** Differential expression analysis of lncRNAs, mRNAs and miRNAs.

**Additional file 7: Table S7.** GO and KEGG pathway analysis of the targets for differentially expressed lncRNAs.

**Additional file 8: Table S8.** Identification of miRNA–mRNA pairs.

**Additional file 9: Table S9.** GO and KEGG pathway analysis of the targets for differentially expressed miRNAs.

**Additional file 10: Table S10.** Phenotypic records of Canadian Large White sows used in this study.

**Additional file 11: Table S11.** Quantitative real-time PCR primer sequences used in this study.

## Data Availability

The datasets generated for this study can be found in the National Center for Biotechnology Information (NCBI) Gene Expression Omnibus (GEO) database, Accession numbers GSE134001 (https://www.ncbi.nlm.nih.gov/geo/query/acc.cgi?acc=GSE134001) and GSE132307 (https://www.ncbi.nlm.nih.gov/geo/query/acc.cgi?acc=GSE132307). The reference genome (Sscrofa10.2) is publicly available in Ensembl repository (ftp://ftp.ensembl.org/pub/release-86/fasta/sus_scrofa/dna/Sus_scrofa.Sscrofa10.2.dna.toplevel.fa.gz).

## Abbreviations

lncRNAs: Long noncoding RNAs
miRNAs: micro RNAs
RNA-seq: RNA sequencing
MREs: miRNA response elements
RT-qRCR: Reverse Transcription Real**-**time Quantitative PCR
ceRNAs: competing endogenous RNAs
TNB: total number of piglets born
RIN: RNA Integrity Number
FPKM: Fragments per Kilo bases per Million reads
PAQR7: Progestin and adipoQ receptor 7
GRIK4: Glutamate receptor, ionotropic,kainate 4
ILF3: Interleukin Enhancer Binding Factor 3
